# Dyslipidemia and Chronic Inflammation Markers Are Correlated with Telomere Length Shortening in Cushing’s Syndrome

**DOI:** 10.1371/journal.pone.0120185

**Published:** 2015-03-23

**Authors:** Anna Aulinas, María-José Ramírez, María-José Barahona, Elena Valassi, Eugenia Resmini, Eugènia Mato, Alicia Santos, Iris Crespo, Olga Bell, Jordi Surrallés, Susan M. Webb

**Affiliations:** 1 Sant Pau Biomedical Research Institute, Endocrinology/Medicine Departments, Hospital de Sant Pau, Universitat Autònoma de Barcelona, Barcelona, Spain; 2 Center for Biomedical Network Research on Rare Diseases (CIBERER Unit 747), ISCIII, Barcelona, Spain; 3 Universitat Autònoma de Barcelona. Department of Genetics and Microbiology and Center for Biomedical Network Research on Rare Diseases (CIBERER Unit 745), ISCIII, Bellaterra, Barcelona, Spain; 4 Hospital Universitari Mutua Terrassa, Endocrinology Department, Terrassa, Barcelona, Spain; 5 Center for Biomedical Network Research on Bioengineering, Biomaterials and Nanomedicine (CIBER-BBN), ISCIII, Barcelona, Spain; Leiden University Medical Centre, NETHERLANDS

## Abstract

**Introduction:**

Cushing’s syndrome (CS) increases cardiovascular risk (CVR) and adipocytokine imbalance, associated with an increased inflammatory state. Telomere length (TL) shortening is a novel CVR marker, associated with inflammation biomarkers. We hypothesized that inflammatory state and higher CVR in CS might be related to TL shortening, as observed in premature aging.

**Aim:**

To evaluate relationships between TL, CVR and inflammation markers in CS.

**Methods:**

In a cross-sectional study, 77 patients with CS (14 males, 59 pituitary-, 17 adrenal- and 1 ectopic-origin; 21 active disease) and 77 age-, gender-, smoking-matched controls were included. Total white blood cell TL was measured by TRF-Southern technique. Clinical data and blood samples were collected (lipids, adrenal function, glucose). Adiponectin, interleukin-6 (IL6) and C-reactive protein (CRP) were available in a subgroup of patients (n=32). Correlations between TL and clinical features were examined and multiple linear regression analysis was performed to investigate potential predictors of TL.

**Results:**

Dyslipidemic CS had shorter TL than non-dyslipidemic subjects (7328±1274 vs 7957±1137 bp, p<0.05). After adjustment for age and body mass index, cured and active CS dyslipidemic patients had shorter TL than non-dyslipidemic CS (cured: 7187±1309 vs 7868±1104; active: 7203±1262 vs 8615±1056, respectively, p<0.05). Total cholesterol and triglycerides negatively correlated with TL (r-0.279 and -0.259, respectively, p<0.05), as well as CRP and IL6 (r-0.412 and -0.441, respectively, p<0.05). No difference in TL according the presence of other individual CVR factors (hypertension, diabetes mellitus, obesity) were observed in CS or in the control group. Additional TL shortening was observed in dyslipidemic obese patients who were also hypertensive, compared to those with two or less CVR factors (6956±1280 vs 7860±1180, respectively, p<0.001). Age and dyslipidemia were independent negative predictors of TL.

**Conclusion:**

TL is shortened in dyslipidemic CS patients, further worse if hypertension and/or obesity coexist and is negatively correlated with increased inflammation markers. Increased lipids and a “low” grade inflammation may contribute to TL shortening and consequently to premature ageing and increased morbidity in CS.

## Introduction

Cushing’s syndrome (CS) due to chronic exposure to endogenous hypercortisolism may be caused by a pituitary adenoma, an adrenocortical tumor or ectopic adrenocorticotropic hormone (ACTH) or corticotropin-releasing hormone (CRH) production [1]. Nevertheless, the most common cause of CS is the use of exogenous glucocorticoids. CS increases cardiovascular risk factors (CVRF), including impaired glucose tolerance, atherosclerosis, hypertension, dyslipidemia, hypercoagulability, obesity, increased visceral adiposity and insulin resistance [2]. This increased visceral adiposity is associated with altered production of adipocytokines, which determines a “low grade” inflammatory state, promoting a cascade of metabolic aberrations leading to permanent cardiovascular risk [3]. Low levels of adiponectin in CS, and increased release of pro-inflammatory adipocytokines and inflammatory markers, like soluble tumor necrosis factor-α receptors (sTNF-R1, sTNF-R2), interleukin-6 (IL6) and C-reactive protein (CRP) [3, 4] also confer an inflammatory state and increased morbidity and mortality observed in CS.

Telomeres are nucleoprotein structures at the end of eukaryotic chromosomes, made up of several thousand repetitive DNA sequences (TTAGGG) coated by capping proteins. They protect the genome from damage providing chromosome stability. Telomeres shorten with repeated cell division, and cells enter senescence followed by apoptosis when a critically short telomere length (TL) is reached [5]. As telomere shortening is approximately the same in different tissues, circulating leukocytes from blood cells are used as easily accessible surrogate tissue for TL assessment when analysing systemic effects of chronic diseases, like cardiovascular disease [6,7]. Even in “nondividing” cells, telomeres are shortened by oxidative stress, which preferentially damages guanine-rich sequences to a greater extent (as found in telomeres) than nontelomeric DNA. Increasing evidence suggests that one critically short telomere may cause a cell to enter senescence regardless of mean TL [8]. This supports that measurement of the proportion of short telomeres in an individual may provide additional information, since short telomeres may be crucial for cellular senescence.

Premature cell senescence and oxidative stress are both cause and consequence of several CVRF and their complications. In humans it is widely accepted that TL is affected by oxidative stress and considered a novel marker of cardiovascular risk [9,10]. An association between TL shortening and age-related human disorders, like type 2 diabetes mellitus (T2DM), poor lipid profile and high blood pressure have been reported [11,12,13]. Also, short telomeres are associated with increased oxidative stress and inflammation biomarkers, such as CRP and IL6 [14]. Increased circulating inflammation markers and adipocytokines are related to leukocyte turnover stimulation and increased reactive oxygen species (ROS), causing cell damage and telomere attrition [15]. In fact, oxidative stress, inflammation and increased cell turnover associated with CRVF are major determinants of accelerated telomere shortening.

Thus, a major issue in telomere research is to understand what factors, in addition to age, influence TL, with its clinical and therapeutic implications. An imbalance of adipocytokine production and higher prevalence of CVRF have been reported in CS compared to controls [3,4]. TL shortening is also observed in inflammatory states and in cardiovascular disease. Based on these previous evidences which relate premature aging with TL shortening on the one hand, and increased cardiovascular risk and inflammatory state with TL shortening on the other hand, we devised our hypothesis. Since CS is also characterized by increased cardiovascular risk (hypertension, dyslipidemia, central obesity, diabetes…), and increased inflammatory state, we hypothesized that TL shortening may in part be behind and contribute to the increased morbidity and features of premature ageing observed in patients with CS. Therefore, we speculated that TL shortening might be involved in this “low grade” inflammatory state and higher prevalence of CVRF in CS, even when hypercortisolism is biochemically cured.

Most studies on TL have been performed in healthy subjects, T2DM, cardiovascular disease or psychiatric conditions. We recently reported no differences in TL in a cross-sectional comparison of CS and controls, but when patients with active CS were evaluated longitudinally after biochemical control, telomere lengthening was observed despite being on average 3 years older [16]. However, no study has reported data on TL in CS related to metabolic or inflammatory state. Thus, our aim was to evaluate the relationship between TL, CVRF and inflammation markers in patients with CS and investigate major determinants of TL.

## Materials and Methods

### Subjects

In this cross-sectional study, patients with CS followed since 1982 were eligible. Adrenal carcinomas were excluded. Seventy-seven CS patients and 77 controls, matched for gender, age and smoking participated. Fourteen were men (18%) and 63 women (82%). Mean age was 48.6±12.8 years. Fifty-nine patients were of pituitary origin (77%), 17 of adrenal origin (adenoma or bilateral macronodular hyperplasia) and in one the origin was unknown (ectopic ACTH secretion of unknown source). Twenty-one (27%) had active disease and 56 (73%) were cured (median time of remission was 3.6 years (IQR 11.6)). Eight with active CS (38%) were treated metyrapone, 6 (29%) with ketoconazole and 3 (14%) with both drugs. Median duration of hypercortisolism was 62 months (IQR 70.5). Since mortality and morbidity risk is increased in CS, even before diagnosis and treatment [17], duration of hypercortisolism was considered as the period between onset of symptoms (as referred by patients) and remission of hypercortisolism (in patients in remission) or the time of current analysis (in active patients). Median period between symptoms onset and biochemical diagnosis of CS was 24 months (IQR 37). Twenty-two patients (29%) had undergone pituitary radiotherapy and 71 (92%) surgery. Fifty-three % (n = 41) were cured after initial treatment without recurrence and 20% (n = 15) were cured after further therapies for recurrent disease. Fifteen cured patients (20%) were adrenal insufficient on substitution with hydrocortisone (mean dose 17.6±3.7 mg, range 10–20). Nine (12%) were GH-deficient (4 replaced with recombinant human GH); 8 women (10%) were gonadotropin-deficient (all on estrogen/progesterone hormone replacement), and 15 (19%) were hypothyroid, 10 due to TSH deficiency and 5 due to primary hypothyroidism (all on L-thyroxine replacement). CS was considered in remission if either adrenal insufficiency was demonstrated (basal morning cortisol < 100 nmol/l [<4μg/dl] and/or undetectable 24-h free urinary cortisol) or morning cortisol suppression (<50 nmol/l, <1.8 μg/dl) after 1 mg dexamethasone overnight was observed. Twenty-five (32%) were on antihypertensive medication, 17 (22%) on statin treatment for dyslipidemia, 12 (16%) were treated with calcium and vitamin-D and 7 (9%) for T2DM.

Seventy-seven controls selected from the blood bank donor’s database or from healthy volunteers recruited among hospital employees were matched for gender, age and smoking status, features known to affect TL. Glucocorticoid exposure, severe and/or acute diseases and severe psychiatric alterations were excluded (however, anxiety and mild depression were not exclusion criteria). Four (6%) were on antihypertensive therapy, 4 (6%) were receiving statin treatment, 3 (4%) were treated with calcium and vitamin-D and 1 (1%) with metformin.

Anthropometry (weight, height, body mass index and waist/hip ratio) was measured in all subjects. Obesity was defined as BMI ≥ 30 kg/m^2^. Increased abdominal circumference was defined as >102 centimeters (cm) in men and >88 cm in women. Hypertension was defined as systolic blood pressure >140 mmHg or diastolic blood pressure >90 mmHg or the use of antihypertensive medications. Dyslipidemia was defined as total cholesterol (TC) >5.8 mmol/l, low-density lipoprotein (LDL) >3.4 mmol/l, triglycerides ≥1.7 mmol/l or treatment with lipid-lowering medication. T2DM was confirmed by fasting glucose >126 mg/dL in two consecutive determinations or glucose 2-hour after an oral tolerance test >200 mg/dL. The presence of metabolic syndrome was defined by the criteria of the National Cholesterol Educational Program (NCEP) Adult Treatment Panel III (ATPIII) [18], as modified by the American Heart Association/National Heart, Lung and Blood Institute [19]. Alcohol consumption was divided into non/mild (intake <110 gr/week for women, <170 gr/week for men), or moderate/severe (intake >110 gr/week for women, >170 gr/week for men).

All participants gave written informed consent to the study, approved by the Comité Ético de Investigación Clínica of the Hospital de la Santa Creu i Sant Pau (Code: 10/031/1070), and provided a blood sample for DNA extraction and fasting blood measurements.

### Methods

Genomic DNA extraction from total leukocytes was performed using an adapted Proteinase K and Phenol protocol [20]. Blood samples were collected in EDTA tubes to reduce DNA degradation. Genomic DNA was isolated from blood buffy coats. The buffy coat and white blood cell pellets were stored at-80°C prior to processing. The white bood cell layers were harvested and digested with buffer containing 0.1 M MgCl2, 0.02 M EDTA, 0.5% SDS, 0.01 M Tris, pH 8.0, and 1 mg/mL of proteinase K at 37°C overnight. Lysates were homogenized by passes through a blunt 20-gauge needle (0.9 mm diameter) at 4ºC temperature and DNA was purified by phenol:chloroform:isoamilic alcohol (25:24:1) extraction, and ethanol precipitation. Genomic DNA was dissolved in Tris-EDTA buffer and quantified by spectrophotometric analysis. The quality of genomic DNA was checked for high molecular weight by 1% agarose gel electrophoresis.

TL measurements were performed by telomere restriction fragment assay (TRF) using the Telo TAGGG Telomere Length Assay Kit (Roche 12209136001); 1 μg DNA was digested with 20 units of Rsal and Hinfl for 2 h at 37ºC. Samples were loaded on a 0.5% Seakem Gold Agarose gel and run for 21 h at 35 V. Gels were treated with HCl, denaturalized and neutralized, and transferred to a nylon membrane by capillarity for 12–18 h. After fixation with UV, hybridization was carried out with a DIG-labeled telomeric probe (3 h at 42ºC). Restriction washes, incubation with anti-DIG-AP antibody and detection by chemiluminiscence was carried out. Images were analyzed with the Quantity One program. TRF mean was calculated using the formula: TRF mean = ∑OD*i*/∑(OD*i*/*Li*), where OD*i* is the chemiluminiscent signal and L*i* is the length of the TRF fragment at position *i* [21]. A control sample, 2 μg of digested DNA derived from a single batch of Hela cells, was run on each gel to minimize interassay variation. The mean TL for Hela cells was 4114 bp with a standard deviation of ±210 base pairs (bp), in the acceptable range of accuracy of the Southern Blot Technique (around 300 bp) [22]. Using the same films as in mean TRF analysis, the proportion of short telomeres (< 5 kb) were calculated in each sample. Total chemiluminiscence intensity of each sample and that below molecular size marker 5 kb were measured. Background was fixed as the signal at the nadir of the low molecular weight region.

Biochemistry and hormone analyses. Fasting samples for routine determinations by standard automated laboratory methods were obtained for glucose, total cholesterol, high (HDL) and LDL cholesterol and triglycerides. Blood counts were performed using automated cell counters. Twenty-four-hour urinary free cortisol was measured with a commercial RIA (Coat-A- Count Cortisol, Siemens) with prior extraction with an organic solvent; intra and interassay coefficients of variation (CV) were 5.1 and 6.4% respectively. Plasma ACTH was measured by chemiluminiscent immunometric assay (Immulite 2000, Siemens Healthcare Diagnostics Products Ltd., Llanberis, UK; intra and interassay CV of 9.5 and 10%). Serum cortisol was measured by electro-chemiluminescent immunoassay (Modular Analytics E170, Roche Diagnostics GmbH, Mannheim, Germany; intra and interassay CV of 1.7 and 2.8%). Adiponectin was determined by ELISA (EZHADP-61K; PromoCell GmbH, Heidelberg, Germany; intra and interassay CV of 3.4 and 5.7%). Serum IL-6 was determined by high sensitivity ELISA (Bender MedSystems GmbH, Vienna, Austria; intra and interassay CV of 6.9% and 8%). Plasma sTNF-R1 and sTNF-R2 were evaluated by solid phase Enzyme Amplified Sensitivity Immunoassays (Biosource Europe S.A., Fleunes, Belgium; intra and interassay CV <8%). Serum CRP was measured by immunoturbidimetric assay (Modular DPE, Roche Diagnostics GmbH, Mannheim, Germany; intra and interassay CV of 2.76 and 4.61%).

### Statistical analysis

A descriptive analysis was performed to verify correct introduction of data in the database. Quantitative data are expressed as mean and SD (Gaussian distribution) or as median (p50) and interquartile range (IQR) (non-Gaussian distribution), and categorical data as percentages. Data distribution was analyzed by the Kolmogorov-Smirnov test. TL variable was normally distributed. Logarithmic transformations were performed where necessary to normalize distribution. Comparison between 2 groups was performed using Student’s t (Gaussian distribution) or Mann-Whitney’s U (non-Gaussian distribution) tests. A Chi-square test was performed for categorical variables. Fisher exact test was performed when appropriate. Pearson’s correlation coefficient was used to estimate linear association between two quantitative variables. Analysis of covariance (ANCOVA) was performed to evaluate TL after adjustment for age (as covariate).

Multivariate linear regression analysis (stepwise) including variables correlated with TL in a univariate analysis and others clinically relevant as potential predictive factors for TL (dependent variable) was performed.

Statistical analyses were performed using the SPSS 21.0 statistical package for Windows (SPSS Inc, Chicago Illinois). Statistical significance was accepted at p<0.05.

## Results

**Fig 1:**
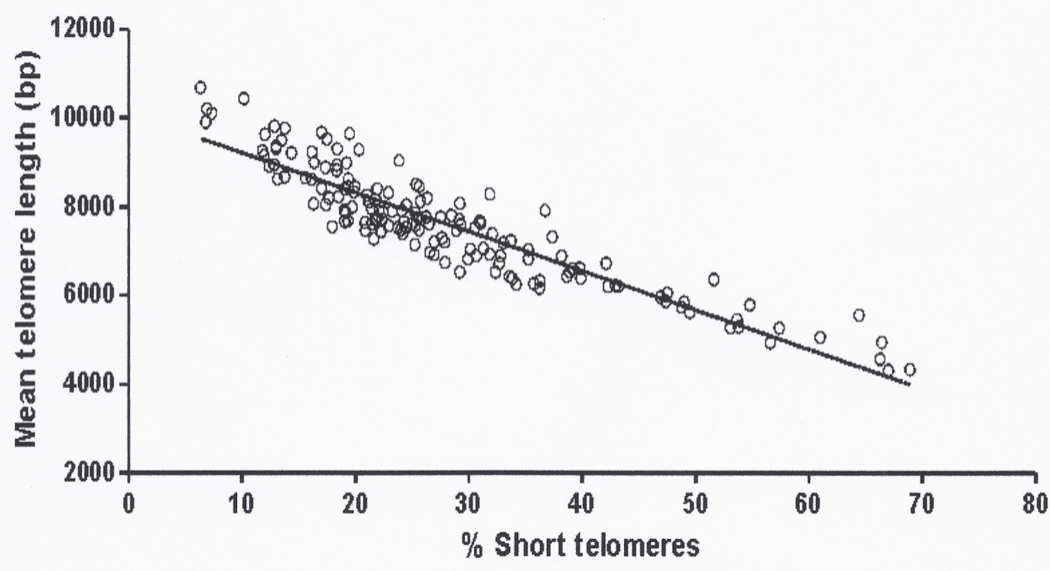
Correlation between mean telomere length and proportion of short telomeres (< 5kb) in the study population (r-0.917, p < 0.001).

Clinical and biochemical characteristics of the subjects included in the study are shown in Table 1. TL declined with age as expected (r = -0.400, p<0.001). Mean TL was strongly correlated with the proportion of short telomeres (<5 kb) (r = -0.917, p < 0.001) (Fig. 1). No differences in TL were observed related to disease activity nor was there any correlation between duration of hypercortisolism and TL (r = -0.082, NS).

**Table 1:** Clinical and biochemical characteristics of patients with Cushing’s syndrome (CS) and controls.

	CS (n = 77)	Controls (n = 77)	p
**Clinical characteristics**			
Age (years)	48.6± 12.8	48.4± 12.6	NS
Smokers (%)	25%	19%	NS
Moderate alcohol consumption (%)	26%	27%	NS
Diabetes mellitus (%)	14%	1%	<0.05
Hypertension (%)	57%	13%	<0.001
Dyslipidemia (%)	46%	20%	<0.05
Osteoporosis (%)	30%	3%	<0.001
Psychiatric history (%)	38%	11%	<0.001
Body mass index (kg/m2)	28 ± 5.6	26.4 ± 4.9	<0.05
Waist to hip ratio	0.92±0.07	0.85±0.07	<0.05
Metabolic syndrome n (%)*	40%	15%	<0.001
**Lipid and metabolic profile** **			
Triglycerides (mmol/liter)	1.2±0.6	1.09±0.7	0.089
Total cholesterol (mmol/liter)	5.4 ± 1.05	5.3±1.1	NS
HDL cholesterol (mmol/liter)	1.5±0.4	1.5±0.3	NS
LDL cholesterol (mmol/liter)	3.5±0.8	3.4±1.1	NS
Lpa (mg/liter)	410.7±451.1	264±310.8	0.06
**Adipocytokines and inflammatory markers**			
	CS (n = 32)	Controls (n = 32)	
Adiponectin (ng/ml)	14.6 ± 6.8	18.6 ± 10	0.053
IL6 (pg/ml)	1.18±2.1	0.37±0.33	<0.001
sTNF-R1 (ng/ml)	1.87±0.69	1.31±0.32	<0.001
sTNF-R2 (ng/ml)	3.71±2.08	3.09±0.91	NS
C-reactive protein (mcg/ml)	0.37±0.26	0.36±0.38	NS

Abbreviations: Lpa: lipoprotein a; sTNF-R1, sTNF-R2: soluble tumor necrosis factor-α receptors; IL6: interleukin-6.

*As described in references 16 and 17.

**49% of dyslipidemic CS patients and 26% of dyslipidemic controls were on lipid lowering medications.

**Fig 2:**
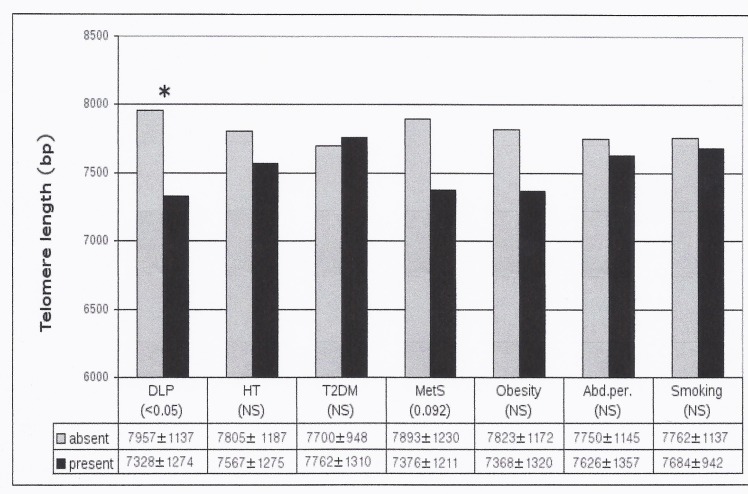
Mean telomere length according to different cardiovascular risk factors after adjustment for age in Cushing's syndrome patients. Abbreviations: bp, base pairs; DLP dyslipidemia, HT hypertension, T2DM Type 2 diabetes mellitus; MetS, metabolic syndrome; abd.per., increased abdominal perimeter.* p<0.05

Mean TL after adjustment for age depending on the presence or absence of CVRF were analyzed (Fig. 2). CS with dyslipidemia had shorter TL than those without (7328±1274 vs 7957±1137, p 0.024). Dyslipidemic CS also had a higher proportion of short telomeres (<5kb) compared to non-dyslipidemic CS patients (31.7±2.2 vs 24.8±2.03%, p 0.029). Patients with CS plus obesity or hypertension or metabolic syndrome showed shorter TL than those without, although these differences lost statistical significance after adjusting for age.

**Fig 3:**
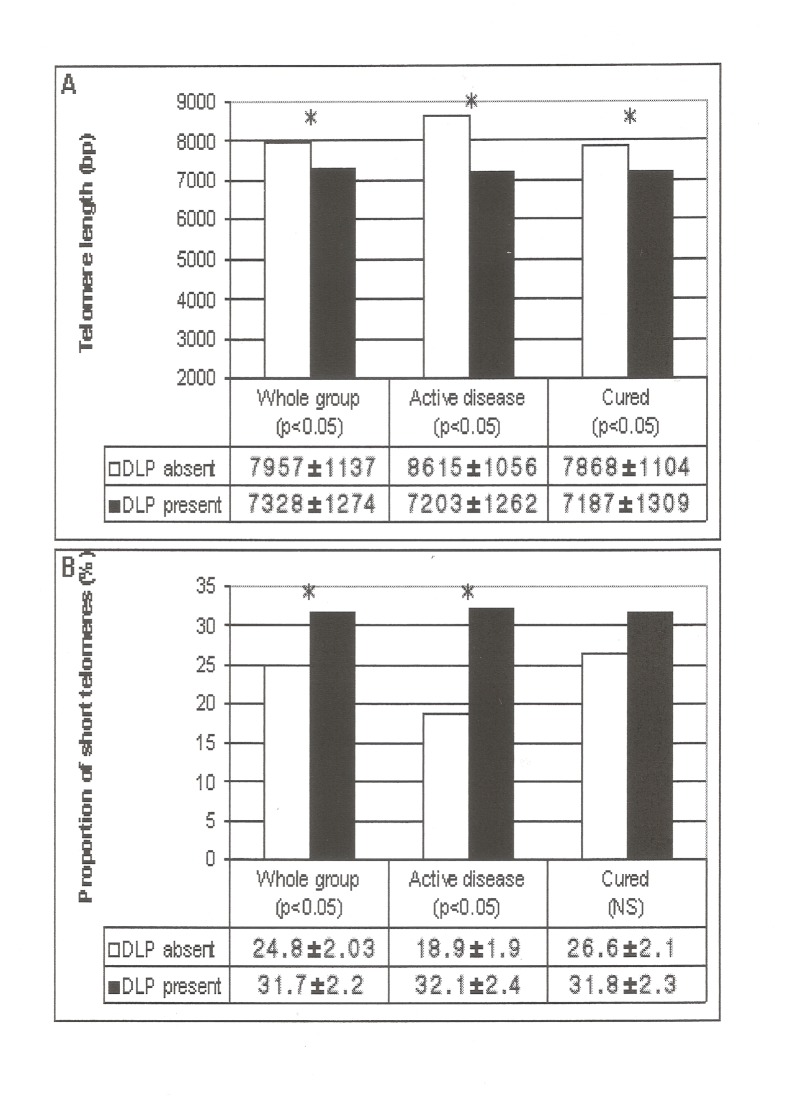
Mean telomere length (A) and proportion of short telomeres (<5kb) (B) in patients with Cushing’s syndrome according to the presence or absence of dyslipidemia (*p < 0.05). Abbreviations: bp base pairs; DLP dyslipidemia.

When cured and active CS were evaluated separately, those with dyslipidemia, independently of being active or in remission of hypercortisolism, presented shorter TL and a higher proportion of short telomeres than those without dyslipidemia (Fig. 3A-B). When clinical characteristics (age, gender, smoking, hypertension, diabetes, activity of disease, cardiovascular disease, obesity, menopausal status) between dyslipidemic and non-dyslipidemic CS patients were compared, to explain shorter TL in dyslipidemic CS patients, the former were older (dyslipidemic 53±11.7 years vs non dyslipidemic 45±12.7 years, p < 0.05) and more frequently obese (49% vs. 34% in non-dyslipidemic CS patients, p < 0.05); these differences persisted after adjustment for BMI and age (7313±1210 vs 7873±1182 bp, p <0.05). We did not observe differences in TL in patients taking or not statin therapy. No differences in other CVRF according to activity of the disease were observed.

**Fig 4:**
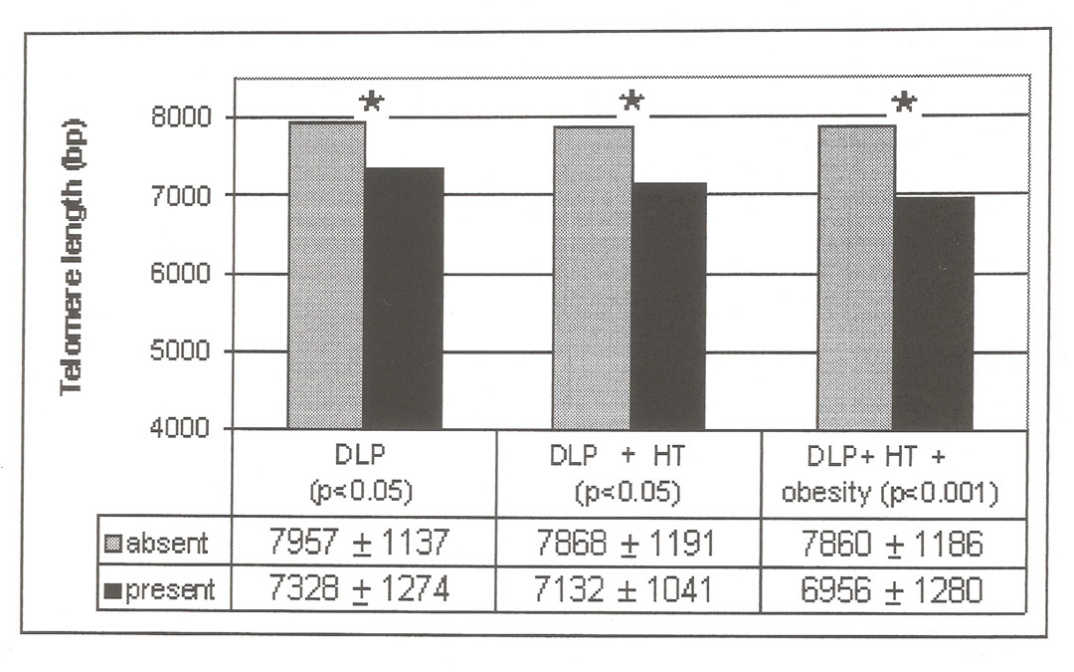
Mean Telomere length in patients with CS with several CVR factors. Dyslipidemic patients (n = 35) compared to those with normal lipids (n = 42); dyslipidemic and hypertensive patients (n = 21) compared to those who did not have both CVR factors (n = 56); patients with dyslipidemia, hypertension and obesity (n = 15) compared to those who did not have three CVR factors (n = 62). Abbreviations: DLP: dyslipidemia; HT: hypertension; bp: base pairs

Twenty-one CS presented both dyslipidemia and hypertension; after adjustment for age and BMI TL was shorter compared to CS patients without dyslipidemia and/or hypertension (7132±1041 bp vs 7868±1191 bp, respectively, p< 0.05). Fifteen CS patients presented with three CRVF (dyslipidemia, hypertension and obesity); TL was shorter compared to those without three concomitant CVRF (6956±1280 vs 7860±1180, respectively, p < 0.001) (Fig. 4). TL did not differ related to disease activity. No differences in TL according to the presence or absence of T2DM, smoking habit and increased abdominal circumference were observed. No differences between TL according to the presence or absence of CVRF (dyslipidemia, hypertension and metabolic syndrome) after adjustment for age and BMI were observed in the control group.

Correlations between TL and dyslipidemic-related parameters (Table 2) in 60 patients not treated with statins showed a negative correlation of total cholesterol and triglycerides with TL (r-0.279 and r–0.259, respectively, p<0.05). No correlations were found with HDL (r-0.236), or with LDL (r-0.05). In 17 dyslipidemic CS patients on statin therapy, no correlations were found with any lipid parameter.

**Table 2:** Correlations of telomere length with lipid profile in patients with Cushing’s syndrome without statin treatment (n = 60).

Parameter	r coefficient	p
Triglycerides	-0.259	< 0.05
Total cholesterol	-0.279	< 0.05
LDL cholesterol	-0.05	NS
HDL cholesterol	-0.236	NS

Abbreviations: LDL low density lipoprotein cholesterol; HDL high density lipoprotein cholesterol.

### Correlations of TL, adipocytokines and inflammation markers

In 32 CS (25 cured, 7 with active disease), evaluation of adipocytokines and inflammation markers was possible (Table 1).

**Fig 5:**
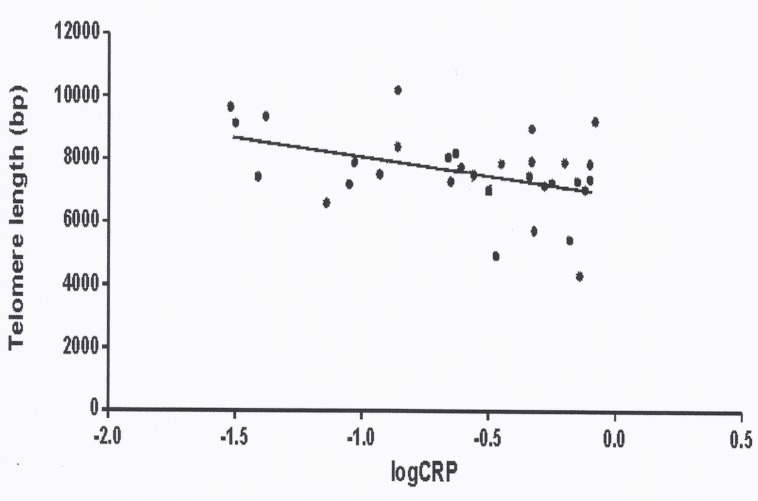
Correlations between C-reactive protein (expressed as logarithm) and telomere length (in base pairs = bp) in patients with Cushing’s syndrome (r-0.412, p 0.019).

A negative correlation between CRP and TL was observed (r-0.412, p = 0.019) (Fig. 5). Also, a negative correlation between IL6 and TL was found (r-0.441, p = 0.016). No other significant correlations were observed between other adipocytokines and TL (adiponectin r 0.131, sTNF-R1 r-0.186 and TNF-R2 r-0.128, NS). The proportion of short telomeres also correlated positively with CRP (r 0.437, p 0.012) and IL6 (r 0.328, p 0.036), but not with adiponectin, sTNF-R1 or sTNF-R2.

### TL determinants

A multiple linear regression analysis to evaluate determinants of TL in CS included age, gender, T2DM, hypertension, dyslipidemia, smoking, obesity, duration of hypercortisolism and disease activity in the model, to find predictors of TL. Age (ß-32, t-3.01, p = 0.004) and dyslipidemia (ß-310, t-2.10, p = 0.030) were the only negative independent predictors of TL (R^2^ 0.21).

## Discussion

Our initial hypothesis was that TL shortening might be involved in the “low grade” inflammatory state and higher prevalence of CVRF in CS, even when hypercortisolism is biochemically cured. For this reason, our approach was to investigate the relationship between TL, classical cardiovascular risk factors and inflammation markers in CS patients. The two main findings are the negative impact of dyslipidemia, further worsened if hypertension and or obesity coexist, and inflammation markers (CRP and IL-6) on TL maintenance. To the best of our knowledge this is the first study to evaluate the relation between individual CVRF and TL in CS.

As expected, TL was inversely correlated with age, as described in much larger populations, supporting the reliability and validity of our results and the methodology used [23]. However, many factors affect TL, both individual and environmental (genetic, epigenetic, socio-economic status, lifestyle, growth factors, etc.), and should be taken into account when interpreting the results.

We found that dyslipidemia after adjusting for age and disease activity and elevations of CRP and IL6 were the main factors negatively related to telomere lengthening in CS, even after controlling for other clinical and metabolic confounders. Other individual CVRF (hypertension, smoking, T2DM, obesity) were not correlated with TL. CS patients with dyslipidemia had shorter TL in all stages (active, in remission or adrenal insufficient after surgery) compared to CS without dyslipidemia. Differences in TL between dyslipidemic and non-dyslipidemic patients persisted after adjustment for BMI (greater in dyslipidemic patients), as suggested by some authors [13, 24, 25]. However, TL shortening was not associated with dyslipidemia in the control group, probably due to the low prevalence of dyslipidemia observed in controls (n = 15), which reduced statistical power and prevented firm conclusions in this group of healthy controls. Additional TL shortening was found in patients with both dyslipidemia and hypertension. Not surprisingly TL was even shorter when these patients were also obese, since excessive adiposity results in a metabolic imbalance, with an increased inflammatory state and oxidative stress, phenomena associated to accelerated telomere shortening [13,14].

Available literature on the relation between TL, lipids and other CVRF is often discordant. Our findings, namely a negative correlation of total cholesterol and triglycerides and TL are consistent with several but not all previous studies (Table 3). Similar to our findings, in a healthy young population at low cardiovascular risk, an inverse correlation between triglycerides and TL was observed [26, 27]. In T2DM patients, an association between shorter TL and oxidative stress was reported [10], as well as an inverse correlation between TL and total cholesterol, LDL-cholesterol, BMI, triglycerides and CRP [12, 14]. However, other studies observed no relation between TL and CVRF in a population without cardiovascular disease [27, 28]. Similar findings were reported in obese children [29], stable coronary artery disease [30] or myocardial infarction [31].

**Table 3:** Studies examining relationships between the telomere system and lipid related parameters.

Study population	Reference	Number of subjects n	Main Findings
**Studies reporting shorter TL with poor lipid profile**			
South Asian T2DM (aged 45 to 60 years)	[12]	142	TL inversely correlated with triglycerides and total cholesterol
T2DM without complications	[14]	97M/96F	TL inversely correlated with BMI, LDL, total cholesterol, HOMA-IR, CRP levels.
Healthy adults	[26]	49M/33F	TL inversely correlated with waist circumference, triglycerides and directly correlated with HDL-cholesterol levels
Healthy adult people	[39]	1917	Higher LDL-cholesterol and CRP levels were observed in the shortest tertile group of TL
**Studies not reporting relations between lipid profile and TL**			
Caucasian T2DM	[10]	569	No correlations were found between total cholesterol, LDL-cholesterol, HDL-cholesterol, triglycerides and TL.
Subjects free of overt CVD	[28]	1218M/ 1291F	No correlations were found between total cholesterol, LDL-cholesterol, HDL-cholesterol, triglycerides and TL.
Patients from Helsinki Businessmen Study	[27]	436 M	No correlations between total cholesterol levels and TL were found in older ages.
T1DM patients	[36]	132	No correlations were found between BMI, LDL-cholesterol, CRP, duration of diabetes and TL
Patients with stable coronary artery disease	[30]	780	No differences in LDL-cholesterol, HDL-cholesterol were observed according to different quartiles of TL.
French obese and non-obese children	[29]	471/322	No correlations were found between total cholesterol, HDL-cholesterol and TL

Abbreviations: TL, telomere length; T2DM, type 2 diabetes mellitus; M, male; F, female; T1DM, type 1 diabetes mellitus; BMI, body mass index; HOMA-IR: homeostasis model assessment of insulin resistance; CRP, C-reactive protein; LDL, low density lipoprotein; HDL, high density lipoprotein; CVD, cardiovascular disease.

We were unable to demonstrate effects of CS activity (active or cured hypercortisolism), hypopituitarism or hydrocortisone replacement on TL. Since statin therapy prevents TL erosion of endothelial progenitor cells in healthy subjects [31], cholesterol lowering medications may preserve or even elongate TL; since our study was not longitudinal, this preservation effect of statins on TL could not be evaluated.

Another interesting finding is the negative correlation between inflammation markers (CRP and IL6) and TL. Similar findings were reported in 2500 healthy Caucasians supporting that chronic systemic inflammation promotes both atherogenesis and telomere attrition [27]. Also in 36 healthy women where optimism and pessimism were evaluated, a strong negative correlation between TL and IL6 was observed in the pessimist state [32]. Another recent study showed that adipocytes under oxidative stress had shortened telomeres, increased mRNA protein expression of IL6 and sTNF, with decreased expression of adiponectin [33]. Adiponectin has anti-atherogenic and anti-inflammatory properties, protective against metabolic phenomena known to accelerate aging. Glucocorticoids inhibit adiponectin secretion [4]; thus, as expected, lower adiponectin was observed in CS compared to matched controls [3]. Interestingly, a correlation has been observed between telomere shortening and hypoadiponectinemia in obesity [34], and we also found a trend, which was not statistically significant, probably due to the limited sample size.

Although the mechanisms involved are unclear, we propose the following hypothesis. Elevated cholesterol and triglycerides are atherogenic, determining repeated mechanical, hemodynamic, and/or immunological injury, increasing cell turnover and production of ROS in certain cells (as in subclinical chronic inflammation) [35]. The link between cholesterol and TL may be through this increased cell damage and turnover, leading cells to their maximum replicative capacity and translating into shortened TL and cell ageing [14]. Unfavourable lipid phenotypes would then determine increased oxidative stress, accelerated senescence and cell aging, which in turn could explain our finding in CS.

Most studies only report mean TL. Increasing evidence suggests that regardless of mean TL, the presence of a few critically short telomeres may cause a cell to enter senescence [36, 37]. Therefore, we measured the proportion of short telomeres. CS patients with dyslipidemia exhibited a higher proportion of short telomeres. Whether short TL imply a higher risk of dyslipidemia or if dyslipidemia hastens shortening of telomeres is currently unknown.

The study has several limitations. Due to its cross-sectional nature causality cannot be inferred, limiting conclusions on the potential relationship between TL and dyslipidemia or inflammatory markers. The sample size, although respectable considering that CS is a rare disease, precludes analysis of different etiological subgroups of CS; neither does it allow controlling for all potential confounders, especially medical treatment during active disease, physical activity, individual variability of possible drug effects on telomere attrition, etc. Additionally, even in individuals of similar age, TL may show inter-individual variability [38]. It would be interesting to evaluate TL in other tissue samples (vascular cells, adipocytes) as we can not ensure that our findings are reproducible in cells of the cardiovascular system, because glucocorticoids induce changes in the immune system. However, this would be even more difficult than obtaining peripheral leukocytes for TL evaluation. Finally, even though most cross-sectional studies on telomere biology and ageing are much larger, large-scale, longitudinal, prospective and well-designed studies in general population are still lacking, so that the influence of different physiological states on TL still have to be elucidated.

In summary, in CS patients TL is shortened in those with dyslipidemia; if obesity and/or hypertension were also present, TL was even shorter than if dyslipidemia was present alone. Furthermore, reduced TL is negatively correlated with increased inflammation markers, suggesting that dyslipidemia and “low” grade of inflammation directly contribute to TL shortening, premature ageing and increased morbidities in CS. Larger prospective series and molecular and cellular functional studies are necessary to confirm these findings and to gain more insight on the pathogenesis of TL shortening in CS.

## Supporting Information

S1 Dataset (XLS)
